# Mitochondrial metabolic rescue in post-COVID-19 syndrome: MR spectroscopy insights and precision nutritional therapeutics

**DOI:** 10.3389/fimmu.2025.1597370

**Published:** 2025-05-23

**Authors:** Li-zhen Chen, Qi Cai, Peng-fei Zheng

**Affiliations:** ^1^ Department of Pharmacy, The First Hospital of Putian City, Putian, Fujian, China; ^2^ College of Environmental and Biological Engineering, Putian University, Putian, Fujian, China; ^3^ School of Medicine, Nankai University, Tianjin, China; ^4^ School of Computer Science and Technology, University of Science and Technology of China, Hefei, Anhui, China

**Keywords:** post-COVID-19 condition, mitochondrial dysfunction, nutrition, magnetic resonance spectroscopy, precision medicine

## Abstract

Post-COVID-19 Condition (PCC), impacting 30–90% of survivors, is characterized by persistent fatigue and metabolic dysfunction, often linked to underlying mitochondrial impairment. This review examines current evidence on mitochondrial-targeted nutrition therapies, with a focus on magnetic resonance spectroscopy (MRS) as a tool for assessing metabolic recovery. Key findings highlight reduced adenosine triphosphate (ATP) production, heightened oxidative stress, and disrupted mitochondrial biogenesis- metabolic abnormalities that closely mirror those seen in chronic fatigue syndromes. While mitochondrial dysfunction is recognized as central, debate continues on whether systemic inflammation or direct viral damage primarily drives these abnormalities. Current evidence supports nutrients, such as, CoQ10, NAC, and creatine for restoring energy metabolism and reducing oxidative stress. MRS biomarkers (τPCr, Qmax), offer valuable tools for monitoring personalized intervention. However, several limitations persist, including variability in nutritional protocols, inconsistencies in MRS methodologies, and limited consideration of microbiome-psychosocial interactions. Most clinical trials focus on short-term outcomes, lacking data on long-term efficacy or stratification based on mitochondrial dysfunction severity. Future research priorities include multi-omics investigations into mitochondrial-epigenetic interactions, the development of targeted antioxidants, and exploration of engineered microbial metabolites. Standardizing MRS protocols, validating composite endpoints, and optimizing nutrient delivery systems require interdisciplinary collaboration. This review advocates for a precision medicine approach, combining MRS-based metabolic profiling with personalized nutritional strategies, to address the multifactorial nature of PCC and advance clinical translation.

## 1 Introduction

Post-COVID-19 Condition (PCC) has emerged as a significant public health concern (1), with a global incidence rates among COVID-19 survivors reported to range between 30% to 90% (2). This condition involves a broad spectrum of symptoms, including pervasive fatigue and diminished exercise tolerance, which notably impair quality of life and daily functioning (3, 4). The multifaceted nature of muscle-related symptoms in PCC has drawn comparisons to chronic fatigue syndrome (CFS), underscoring a potentially shared pathophysiology, particularly mitochondrial dysfunction (5, 6).

Mitochondria play a crucial role in energy metabolism, especially in muscle tissue, and disruptions in mitochondrial integrity during acute viral infections may contribute to the muscle fatigue experienced in PCC (6, 7). This dysfunction leads to decreased ATP synthesis and increased production of reactive oxygen species (ROS), which exacerbates oxidative stress, potentially contributing to further cellular damage (5). Emerging evidence also suggests that the lingering fatigue, often observed in PCC, mirrors symptoms seen in aging and neurodegenerative diseases, implying overlapping mechanisms of mitochondrial injury across different conditions including myalgic encephalomyelitis/CFS (ME/CFS) (8, 9, 10).

Discrepancies in research findings on the pathophysiology of PCC highlight the need for deeper investigation. While some studies suggest that the inflammatory response triggered by SARS-CoV-2 may heavily influence the persistence and severity of symptoms (11, 12), others indicate that cognitive impairments in PCC may stem from subtle neurochemical changes linked to mitochondrial dysfunction than from systemic inflammation (4, 13). This underscores the complex and multifaceted nature of PCC, suggesting that no single cause fully accounts for its diverse manifestations seen among patients. Comprehensive understanding of these interrelated mechanisms is crucial for developing targeted mitochondrial nutritional therapies (MNTs) aimed at restoring mitochondrial function and alleviating associated symptoms (14, 15).

The exploration of MNT holds great promise for managing PCC. Considering the evident mitochondrial dysfunction observed in many patients, targeted supplementation may not only help improve energy metabolism, but could also mitigate oxidative stress and restore overall cellular health, potentially supporting recovery from the debilitating effects of prolonged COVID-19 symptoms (7, 16). Increased interdisciplinary collaborations are necessary to consolidate these findings and effectively advance therapeutic strategies to address the multifactorial nature of PCC.

## 2 Mitochondrial dysfunction in PCC

Mitochondrial dysfunction is closely linked to a reduced capacity for synthesizing ATP, which is vital for cellular energy. COVID-19 infection can alter phosphocreatine (PCr) metabolism, a key process facilitating ATP production, ultimately manifesting as profound fatigue and diminished physical capacity in recovering patients (16, 17). Additionally, a well-documented similarity exists between the fatigue profiles of post-COVID-19 patients and those suffering from CFS, suggesting similar underlying mitochondrial dysfunctions that may compromise energy production (18, 19).

Beyond the disruption of energy metabolism, oxidative stress plays a pivotal role in exacerbating mitochondrial dysfunction in PCC. Excessive ROS production following SARS-CoV-2 infection is considered a key pathological mechanism, inducing a cascade of oxidative damage that impairs mitochondrial function, ultimately creating a vicious cycle where decreased mitochondrial respiration leads to further oxidative stress (20, 21). Additionally, imbalances in the antioxidant defense systems, such as reduced glutathione levels, have been observed, particularly among older patients, linking heightened oxidative stress to poorer clinical outcomes (22, 23). A study has further demonstrated that patients with post-acute sequelae of COVID-19 exhibited compromised mitochondrial-nuclear communication and a pronounced shift towards reliance on glycolysis instead of oxidative phosphorylation (24). While this shift may serve as an adaptive response to ongoing oxidative stress, it ultimately contributes to the fatigue and neurological symptoms commonly reported in PCC (25).

Recent evidence has also highlighted that mitochondrial dysfunction is accompanied by disruptions in signaling pathways governing mitochondrial-nuclear interactions. The resultant impaired communications can lead to dysregulation of key metabolic processes, further amplifying oxidative stress responses and perpetuating a detrimental cycle of cellular function (24, 26). Thus, although there is consensus regarding the critical roles of impaired ATP synthesis and heightened oxidative stress in PCC, the intricacies of their interplay and the resulting clinical manifestations remain areas of active investigation and debate.

## 3 MRS in PCC: Mitochondrial bioenergetics and recovery

Magnetic resonance spectroscopy (MRS) is emerging as an innovative tool for dynamically evaluating mitochondrial function and nutritional recovery in individuals with PCC. This technique, particularly utilizing ^1^H and ^31^P-MRS, enables real-time monitoring of metabolite fluctuations during exercise and recovery, with a specific focus on muscle tissues such as the gastrocnemius (27, 28). Key metrics derived from this analysis include Qmax, representing the maximal oxidative flux, and τPCr, the PCr recovery time constant. These parameters offer valuable insights into mitochondrial bioenergetics and muscle recovery capacity, both critical to understanding the complexities of PCC (22, 28).

Clinical validation of MRS findings has revealed significant correlations between elevated resting PCr levels and reduced Qmax in patients post-COVID-19 hospitalization. Such results suggest that optimizing these metrics could be pivotal in addressing the observed fatigue and malaise commonly associated with PCC (28, 29). Elevated resting PCr levels, in particular, may reflect a compensatory reliance on anaerobic energy pathway due to impaired oxidative metabolism, a characteristic frequently observed in long COVID patients (22). However, challenges remain in aligning advanced MRS data with symptom scoring systems, underscoring an urgent need for standardized protocols that effectively integrate MRS findings into routine clinical evaluation of PCC (27, 28).

Emerging evidence also highlights the dual role of malnutrition in both worsening PCC symptoms and impeding the recovery process. Nutritional deficiencies have been identified to significantly increase post-COVID-19 syndrome risk, especially in vulnerable populations such as cancer patients (30–32). Poor nutritional assessment and management during the acute phase of a COVID-19 infection can lead to prolonged muscle loss and functional decline, emphasizing the importance of comprehensive nutritional support throughout recovery phases (33–35). This aspect is particularly relevant in the context ofMRS-guided interventions, as optimizing nutritional intakeis critical for restoring mitochondrial function, a factor that has been associated with improved clinical outcomes in PCC rehabilitation (29, 36). However, the literature also indicates conflicting results regarding the effectiveness of various post-COVID nutritional strategies, particularly in relation to the choice of supplements and the timing of administration within MRS-guided rehabilitation protocols (37, 38).

The application of MRS to evaluate metabolic and nutritional dynamics in PCC shows significant potential in clinical practice. While the clinical validation of MRS-derived parameters like Qmax and τPCr is promising, further research is needed to integrate these insights with nutritional strategies that facilitate optimal recovery and effective symptom management.

## 4 MNT in PCC: MRS-informed strategies for metabolic recovery

The assessment of targeted MNT for individuals suffering from PCC emphasizes the critical insights gained through MRS evaluations. MRS data has been instrumental in identifying specific metabolic abnormalities commonly observed in PCC patients, serving as a guide for tailoring nutritional interventions. For instance, PCr insufficiency observed in these patients suggests a need for creatine supplementation, which directly aids in replenishing energy substrates and enhances cellular metabolism under oxidative stress (29). This aligns with findings suggesting that direct supplementation of PCr precursors can be beneficial in addressing energy deficits characteristics of post-viral syndromes (39).

In addition, reduced oxidative flux has been documented in PCC patients. In this context, nutrients such as Coenzyme Q10 and lipoic acid, have gained attention for their roles in enhancing the efficiency of the electron transport chain and promoting cellular respiration (40). Such supplementation has shown promise in improving mitochondrial function, thereby addressing a central metabolic deficiency noted in patients with long COVID (41). Furthermore, the excessive accumulation of ROS poses a significant challenge in PCC, as it contributes to ongoing mitochondrial dysfunction. Antioxidants like N-acetylcysteine (NAC) and vitamins C and E are known for their ability to neutralize free radicals and regenerate glutathione, thereby offering protection against oxidative damage while supporting mitochondrial integrity (3).

The inhibition of mitochondrial biogenesis in long COVID highlights the importance of targeted nutritional strategies. Nutrients such as pyrroloquinoline quinone (PQQ) and omega-3 fatty acids activate the peroxisome proliferator-activated receptor gamma coactivator 1-alpha (PGC-1α) pathway, a central regulator of mitochondrial biogenesis (42). By promoting the formation of new mitochondria and enhancing overall metabolic flexibility, these nutrients could play a vital role in facilitating recovery in PCC patients.

Emerging research also points to the potential of branched-chain amino acids (BCAAs) in regulating mitochondrial protein synthesis, in muscle tissues an area of particular relevance given the muscle wasting frequently seen in long COVID (43). Furthermore, growing interest surrounds the gut–mitochondria axis, with probiotics and prebiotics being explored for their ability to modulate gut microbiota and, in turn, influence mitochondrial function and overall metabolic health (2). This holistic approach acknowledges the interconnected nature of biological systems, and is crucial for the rehabilitation of patients recovering from COVID-19.

Taken together, the integration of targeted mitochondrial nutrient screening and supplementation holds promise for mitigating the metabolic dysfunctions associated with post-COVID-19 conditions, potentially improving patient outcomes and recovery trajectories. Nonetheless, future investigations are warranted to resolve inconsistencies in the current literature surrounding these interventions and to optimize therapeutic strategies.

## 5 MRS-guided nutritional interventions for PCC recovery

The development of a targeted MNT regimen for individuals suffering from PCC necessitates a detailed understanding of the biochemical pathways involved in mitochondrial dysfunction. MRS has emerged as a pivotal tool for investigating metabolic alterations within the mitochondria, offering valuable insights into energy metabolism and oxidative stress markers. Recent studies have identified several core metabolic abnormalities in PCC patients, including PCr deficiency, impaired oxidative phosphorylation, elevated ROS, and disrupted mitochondrial biogenesis (44).

PCr insufficiency is particularly intriguing, as it implies a potential need for direct supplementation of creatine or PCr precursors. Creatine plays a central role in cellular energy metabolism by acting as a buffering agent for ATP and modulating energy flux within cells. Enhancing PCr levels through supplementation may help restore ATP availability and improve mitochondrial function. Complementing this strategy, compounds such as Coenzyme Q10 (CoQ10) may enhance the efficiency of the electron transport chain, potentially mitigating oxidative stress (45). Similarly, lipoic acid has garnered attention for its dual role in supporting mitochondrial oxidative phosphorylation and functioning as an antioxidant to neutralize excess ROS.

The accumulation of ROS remains a significant challenge in PCC, prompting the use of antioxidants such as NAC and vitamins C and E, which neutralize free radicals and promote glutathione regeneration, a key antioxidant in cellular defense (46, 47). Other promising compounds for restoring mitochondrial function in PCC patients include PQQ and omega-3 fatty acids. PQQ serves as a cofactor that enhances mitochondrial biogenesis through the PGC-1α pathway and possesses antioxidant properties (48). Likewise, omega-3 fatty acids have shown potential to activate PGC-1α, thereby enhancing mitochondrial resilience and adaptability (49).

Emerging candidates for targeted mitochondrial nutrient interventions also encompass BCAAs, which are posited to promote mitochondrial protein synthesis, as along with prebiotics and probiotics that may improve the gut-mitochondria axis (50). These nutritional approaches offer a comprehensive strategy for managing PCC by addressing both mitochondrial deficits highlighted in MRS assessments and broader aspects of cellular health and oxidative stress resilience.

The integration of MRS-guided nutritional interventions offers promise for addressing the unique challenges of PCC. A multifaceted approach, incorporating creatine, CoQ10, lipoic acid, NAC, PQQ, omega-3 fatty acids, BCAAs, and gut microbiome modulators, combines biochemical insights with clinical application. By targeting the cellular energy impairments identified through MRS, this strategy could help restore mitochondrial function, support recovery, and improve the quality of life for PCC patients.

## 6 MRS-guided stratification and personalized nutrition for PCC

MRS can classify patients into groups reflecting mild, moderate, or severe dysfunction through quantitative metrics such as Qmax thresholds. This stratification serves as a crucial foundation for designing personalized therapies that address specific metabolic deficits associated with PCC (30, 38).

One promising avenue within these personalized nutritional interventions is the use of pre-exercise nitrate supplementation, particularly from sources such as beetroot. Nitrate is known to enhance blood perfusion and may improve both exercise performance and recovery by optimizing mitochondrial function. Research suggests that organic nitrates help maintaining vascular function, thereby supporting endothelial health and improving exercise tolerance (51, 52). This approach emphasizes the importance of vascular health in mitigating exercise intolerance commonly seen in this population.

Additionally, the role of nighttime melatonin supplementation offers a compelling strategy for enhancing mitophagy, a critical process for mitochondrial quality control. Melatonin is recognized for its antioxidative properties and may help reduce mitochondrial oxidative stress, enhancing mitochondrial performance during post COVID-19 recovery phases (52, 53). Enhancing autophagic pathways can mitigate cellular damage, contributing to better metabolic health, making melatonin particularly relevant for PCC (52, 54).

Despite the promise of these interventions, the research landscape remains complex and, at times, contradictory. For instance, although dietary supplements like alpha-ketoglutarate have demonstrated metabolic benefits in various settings, evidence for their efficacy in PCC remains inconsistent (54, 55). As such, while MRS-guided stratification and targeted nutritional interventions represent a paradigm shift towards personalized treatment, further research and clinical trials are essential to reconcile discrepancies and validate proposed therapeutic benefits across diverse patient populations (56, 57).

Design of personalized nutritional intervention plans, informed by MRS-guided stratification, holds great promise for enhancing recovery in patients suffering from PCC. The integration of targeted supplementation strategies, such as nitrate and melatonin, can potentially rehabilitate mitochondrial function and improve overall health outcomes. Nonetheless, a rigorous appraisal of existing literature and ongoing clinical evaluations are essential to navigate the complexities and ensure the safe and effective implementation of these nutritional therapies.

## 7 Challenges, multi-omics integration, and emerging translational strategies

The clinical translation of MNT for PCC faces several technical challenges. Limited availability of MRS equipment and lack of standardized protocols across clinical settings lead to variability in data and treatment outcomes (58). Additionally, assessing nutrient bioavailability is complicated by individual physiological factors, dietary patterns, and interactions with medications, which introduce further variability in therapeutic efficacy (59, 60).

Assessment methods used to evaluate the efficacy of mitochondrial interventions exhibit their own set of challenges. One key issue is the need for composite endpoint settings that integrate multiple evaluation metrics such as MRS parameters, the six-minute walk test, and fatigue scales. While integrating these outcome measures could enhance the granularity of results, it also introduces complexity in interpretation and standardization (61). The variability across these assessments also raises the bar for establishing causal relationships between nutritional therapy and clinical improvements. Effective evaluation strategies thus require an interdisciplinary approach that synthesizes expertise from clinical nutrition, pharmacology, and systems medicine to customize interventions for individual patient profiles (62, 63).

Psychosocial factors further influence patient compliance and motivation in utilizing nutritional therapies. There is a growing need for robust frameworks that incorporate such variables into treatment designs. As evidence shows, understanding patients’ lifestyle and psychosocial contexts plays a pivotal role in ensuring compliance and optimizing outcomes (64, 65). Future clinical studies must embrace technologies that facilitate real-time monitoring of patient compliance and feedback, allowing for dynamic adjustments to nutritional interventions based on evolving needs.

Looking forward, research in MNT for PCC should adopt a multi-omics integration approach, combining metabolomics with insights from epigenetics, genomics, transcriptomics, and proteomics. For instance, examining urinary tricarboxylic acid (TCA) cycle intermediates can enrich our understanding of metabolism in the context of nutritional therapy post-COVID-19. Such investigations may elucidate how mitochondrial dysfunction correlates with epigenetic modifications during viral infections and subsequent recovery phases (66). Such multi-layered analyses can uncover novel biomarkers of therapeutic efficacy and pave the way for truly personalized nutrition interventions (67).

The development of novel mitochondria-targeted nutrients also represents a promising frontier. Mitochondria-targeted antioxidants, such as 10-(6’-plastoquinonyl)decyltriphenylphosphonium (SkQ1), have shown potential in counteracting oxidative stress associated with PCC. These targeted interventions can enhance cellular resilience and functionality, thereby supporting the mitochondrial pathways essential for recovery from long-term COVID-19 symptoms (68). In parallel, engineered microbial metabolites, especially butyrate analogs, are gaining recognition for their anti-inflammatory properties and their role in maintaining metabolic health and gut–mitochondria homeostasis after SARS-CoV-2 infection (69, 70).

Effective clinical translation of these exciting avenues into therapy necessitates extensive explorations into their bioavailability, interaction with host systems, and optimal delivery methods. Emerging evidence indicates that encapsulation of bioactive compounds using advanced nanocarrier systems significantly improves their bioactivity by improving their solubility and stability (71, 72).

Equally important is exploring microbiome modulation in relation to mitochondrial health. Probiotics and prebiotics could enhance mitochondrial function by improving nutrient absorption and metabolite generation, which directly impacts mitochondrial performance (73, 74). Investigating these interactions could unveil foundational mechanisms that reshape the future of nutritional therapy landscape.

Translating research into clinical practice will depend on substantiating the links between mitochondrial health and systemic disease processes. Understanding how nutritional interventions influence mitochondrial dynamics could provide insights for developing targeted, evidence-based therapies for PCC. Advancing beyond traditional nutrient roles and embracing systems biology will be essential for bridging research with real-world clinical solutions. The technical challenges surrounding MNT’s application reveal both obstacles and opportunities for clinical advancement. With a commitment to systematic, evidence-based research, integrating multifaceted evaluations and innovative nutritional strategies has the potential to significantly enhance recovery and foster sustainable health improvements in PCC.

## 8 Conclusion

Emerging evidence highlights mitochondrial dysfunction as a central mechanism in PCC, characterized by impaired ATP synthesis, increased oxidative stress, and disrupted mitochondrial-nuclear signaling, which drive persistent fatigue and metabolic derangements in 30-90% of survivors. While nutrients like CoQ10, NAC, and creatine show potential in restoring energy metabolism and reducing oxidative damage, critical gaps remain. These include heterogeneous nutritional protocols, inconsistent MRS methodologies, and a lack of long-term efficacy data, all of which limit clinical translation. Additionally, existing studies focus primarily on biochemical parameters, often neglecting microbiome-psychosocial interactions and failing to stratify patients by the severity of mitochondrial dysfunction, hindering personalized interventions. Future research should prioritize integrating multi-omics approaches to explore mitochondrial-epigenetic crosstalk, standardizing MRS biomarkers (e.g., tPCr, Qmax) for dynamic metabolic profiling, and developing targeted nutrient delivery systems tailored to individual metabolic phenotypes. Interdisciplinary collaboration is crucial to enhancing precision nutrition strategies, bridging mechanistic insights with scalable clinical solutions, and ultimately improving the quality of life for millions affected by PCC.
